# Synthesis and Characterization of Syndiotactic Polystyrene-Polyethylene Block Copolymer

**DOI:** 10.3390/polym11040698

**Published:** 2019-04-16

**Authors:** David Hermann Lamparelli, Vito Speranza, Isabella Camurati, Antonio Buonerba, Leone Oliva

**Affiliations:** 1 Dipartimento di Chimica e Biologia “*Adolfo Zambelli*”, Università degli Studi di Salerno, Via Giovanni Paolo II, 84084 Fisciano (SA), Italy; dlamparelli@unisa.it; 2 Dipartimento di Ingegneria Industriale, Università degli Studi di Salerno, Via Giovanni Paolo II, 84084 Fisciano (SA), Italy; vsperanza@unisa.it; 3 Centro Ricerche G. Natta, Basell Polyolefins, P.le G. Donegani 12, I-44100 Ferrara, Italy; Isabella.Camurati@lyondellbasell.com; 4 C. da Centro Agricolo 27, 75015 Pisticci, Italy

**Keywords:** syndiotactic polystyrene, poly-insertion catalysis, block copolymer, thin film morphology

## Abstract

The direct synthesis of syndiotactic polystyrene-block-polyethylene copolymer (sPS-*b*-PE) with a diblock structure has been achieved. The synthetic strategy consists of the sequential stereocontrolled polymerization of styrene and ethylene in the presence of a single catalytic system: cyclopentadienyltitanium(IV) trichloride activated by modified methylaluminoxane (CpTiCl_3_/MMAO). The reaction conditions suitable for affording the partially living polymerization of these monomers were identified, and the resulting copolymer, purified from contaminant homopolymers, was fully characterized. Gel permeation chromatography coupled with two-dimensional NMR spectroscopy COSY, HSQC, and DOSY confirmed the block nature of the obtained polymer, whose thermal behaviour and thin film morphology were also investigated by differential scanning calorimetry, powder wide angle x-ray diffraction, and atomic force microscopy.

## 1. Introduction

Syndiotactic polystyrene (sPS) is one of the most significant achievements of homogeneous polyinsertion catalysis [1,2]. Besides its employment as engineering plastic [3] thanks to a high melting temperature combined with favourable crystallization kinetics, sPS appears also as a smart polymer due to its nanoporous crystalline structure [4,5,6]. In this respect, many studies have been performed to disclose its ability to selectively clathrate small molecules in order either to remove the undesirable ones from the environment or to release the useful ones into a particular context [7,8]. The permeability of the sPS towards small molecules also suggests interesting applications of this material as support for catalysts [9].

On the other hand, polyinsertion homogeneous catalysis has opened the door for copolymerizing ethylene and styrene to produce chain architectures that are very different both in terms of monomers sequence and stereoregularity [10,11,12,13,14,15,16,17,18,19,20,21,22,23]. Recently, some of us succeeded in the synthesis of an ethylene-styrene block copolymer with a isotactic styrene sequence joined to an ethylene-alt-styrene isotactic sequence [24]. The key to this achievement is living polyinsertion, which can be obtained even with traditional catalysts by working at a low temperature. A similar result was reported by Chu et al. by copolymerizing ethylene and styrene at 50 °C to obtain block copolymer with syndiotactic styrene sequences and short polyethylene segments, while other researchers succeeded in the synthesis of ethylene-styrene copolymers with syndiotactic styrene blocks and minor polyethylene segments with different catalysts [13,25,26].

In light of the significant interest in linear block copolymers, for their ability to give ordered nanostructures [27,28,29], we focussed our efforts on the synthesis of an ethylene-styrene block copolymer with syndiotactic regularity of the styrene sequence and a relevant polyethylene sequence. The catalyst we chose was cyclopentadienyltitanium(IV) trichloride (CpTiCl_3_) activated by modified methylalumoxane (MMAO), one of the more active catalysts for the syndiotactic poly-insertion of the styrene [30]. The same catalyst works in ethylene styrene copolymerization, producing a pseudorandom copolymer in mixture with syndiotactic styrene homopolymer [21] at 50 °C, and was also employed in references [25,26].

## 2. Experimental Section

### 2.1. General Procedures and Materials

Air- and moisture-sensitive operations were carried out in nitrogen atmosphere using standard Schlenk techniques and an MBraun glovebox. Dry solvents were freshly distilled before use. Toluene was kept under reflux in the presence of sodium for 48 h and then distilled in atmosphere of nitrogen. Styrene (99%; Sigma-Aldrich) was purified by stirring overnight in presence of calcium hydride and distillation under reduced pressure. Modified methylaluminoxane (MMAO, 7 wt % of aluminium in toluene; Sigma-Aldrich) was dried in the form of a white powder by removing in vacuo the solvent. Cyclopentadienyltitanium(IV) trichloride (97%; Sigma-Aldrich), ethylene (Linde, Dublin, Ireland), deuterated solvents, and other reagents available from commercial suppliers, unless otherwise stated, were used without further purification. 

### 2.2. Instruments and Measurements

NMR spectra were acquired on Bruker Avance spectrometers (250, 300, 400, or 600 MHz for ^1^H). Chemical shifts (δ) are reported in parts per million (ppm) and referenced using the residual solvent peaks in ^1^H NMR spectra at 7.27 ppm for CDCl_3_ and 5.80 ppm for 1,1,2,2-tetrachloroethane-*d*_2_ (TCE-*d*_2_) and in ^13^C NMR spectra at 77.23 ppm for CDCl_3_ and 73.78 ppm for TCE-*d*_2_. Diffusion-ordered spectroscopy (DOSY) experiments were performed on a Bruker Avance 600 spectrometer equipped with a 5 mm PABBO 600S3 BBF-H-D-OS-Z-SP probe. The standard Bruker pulse program, ledbpgp2s, employing a double stimulated echo sequence and LED, bipolar gradient pulses for diffusion, and two spoil gradients was utilized. The pulse gradients (g) were increased from 5 to 95% of the maximum gradient strength in a linear ramp with a total experiment time of 45 min. Diffusion times were 2500 ms, the eddy current delay was 5 ms, the gradient recovery delay was 0.2 ms, and the gradient pulse was 1000 ms. Individual rows of the quasi-2D diffusion databases were phased and baseline corrected. After Fourier transformation and baseline correction, the diffusion dimension was processed with the software Topspin3.2 and Diffusion from Bruker. Diffusion coefficients, processed with a line broadening of 2 Hz, were calculated by Gaussian fits with the T1/T2 software of Topspin3.2. Molecular weights were calculated using a polystyrene standard for SEC (*vide infra*) with molecular weight of 42.6 kDa and PDI of 1.04 as internal standards, according to the procedure reported in literature [31]. Molecular weight parameters (M*_n_* and M*_w_*) and molecular weight distributions for all the samples were measured by using a SEC-IR apparatus from PolymerChar (Valencia, Spain), which was equipped with a column set of four PLgel Olexis mixed-bed (Polymer Laboratories) and an IR5 detector (PolymerChar). The dimensions of the columns were 300 × 7.5 mm, and their particle size was 13 µm. The mobile phase flow rate was kept at 1.0 mL/min. All the measurements were carried out at 150 °C. Solution concentrations were 1.5 mg/mL (at 150 °C), and 0.3 g/L of 2,6-diterbuthyl-*p*-chresole was added to prevent polymer degradation. For SEC calculation, a universal calibration curve was obtained using 12 polystyrene (PS) standard samples supplied by PolymerChar (molecular weights ranging from 266 to 1,220,000 Da). A third order polynomial fit was used for interpolating the experimental data and obtaining the relevant calibration curve. Data acquisition and processing was done by using Empower 3 (Waters). The Mark–Houwink relationship was used to determine the molecular weight distribution and the relevant average molecular weights: *K* values were *K_PS_* = 1.21 × 10^−4^ dL/g and *K_PE_* = 4.06 × 10^−4^ dL/g for polystyrene (PS) and polyethylene (PE), respectively, while the Mark–Houwink exponents *α* = 0.706 for PS and *α* = 0.725 for PE were used. For PS/PE copolymers, as far as the data evaluation is concerned, it was assumed for each sample that the composition was constant in the whole range of molecular weight, and the *K* value of the Mark–Houwink relationship was calculated using a linear combination (*K*_PS/PE_ = X_PS_*K*_PS_ + X_PE_*K*_PE_), where *K_PS/PE_* is the constant of the copolymer and *x_PS_* and *x_PE_* are the PS and the PE weight relative amount with *x_PS_* + *x_PE_* = 1. Wide-angle x-ray diffraction (WAXD) diffractograms were obtained in reflection using an automatic Bruker D8 diffractometer using Ni-filtered Cu Kα radiation; the sample of Figure 3c in a sealed vial under vacuum was kept for 15 min at 270 °C and then poured into liquid nitrogen before the diffraction. Differential scanning calorimetry (DSC) measurements were carried out in nitrogen on a TA Instrument DSC Q20 calorimeter (heating and cooling rate of 10 °C/min). Atomic force microscopy (AFM) images of polymer thin films were collected in air with a Dimension V microscope coupled with a Bruker Nanoscope V controller (Santa Barbara, CA, USA) operating in tapping mode (TM). The polymers were dissolved at 130 °C in tetrachloroethane (TCE) to afford clear solutions that were deposed (30 μL) onto glass slides at 120 °C and analysed by atomic force microscopy operating in tapping mode (TM-AFM). The thickness of the films (0.3–1 μm) was estimated by TM-AFM height measurements of scratches made by a sharp needle on the films. The TM-AFM micrographs were processed with the Nanoscope Analysis v1.40 r2sr1 software from Bruker.

### 2.3. Typical Procedure for Ethylene-Styrene Copolymerization

**Scheme 1:**
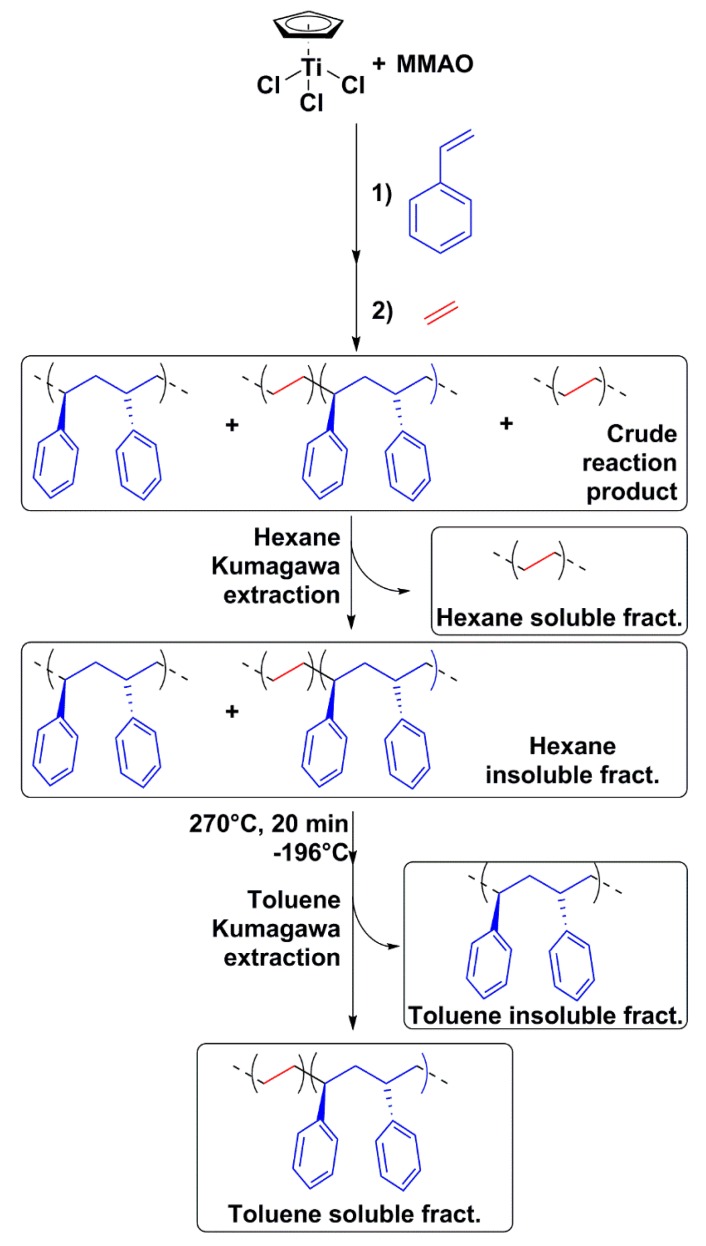
Synthesis and purification of syndiotactic polystyrene-block-polyethylene copolymer (sPS-*b*-PE) block copolymer.

The copolymerization has been realized in a glass tube inside the stainless-steel autoclave (100 mL high-pressure Roth^®^ laboratory autoclave model I) equipped with magnetic stirrer and pressure gauge and thermostated at 0 °C. At first, a solution of toluene (10 mL), styrene (10 mL), CpTiCl_3_ (20 mg), and MMAO (650 mg) was kept to react under nitrogen for a time of 10 min; subsequently, constant ethylene pressure of 2.0 MPa replaced that of nitrogen, and the system remained under stirring for further 180 min. Finally, the gas was vented off, the solution was poured into acidified methanol, and the polymer was collected by filtration and drying in vacuo (Yield: 950 mg). The copolymer fraction has been recovered by sequential extractions in Kumagawa-type extractor (see Scheme 1) with hexane and toluene. The amorphization of the hexane insoluble fraction, by treatment of the solid at 270°C for 20 min and quenching in liquid nitrogen, improves the subsequent extractive processes. The fractions collected were as follows: hexane soluble fraction (30 mg), hexane insoluble-toluene insoluble fraction (530 mg), and hexane-insoluble-toluene-soluble fraction (250 mg; S/E molar ratio = 73:27). 

## 3. Results and Discussion

### 3.1. Synthesis and Characterization of sPS-b-PE Copolymer

In order to achieve the block structure, a solution of toluene, styrene, CpTiCl_3_, and MMAO was reacted under nitrogen at 0 °C for 10 min; subsequently, ethylene at the pressure of 2.0 MPa replaced the nitrogen, and the system remained under stirring and continuous ethylene feed for further 180 min. Finally, the gas phase was vented off; the solution was poured into acidified methanol; and the polymer was collected by filtration, dried, and finally fractionated through sequential extractions in Kumagawa-type extractor (see Scheme 1). It is worth noting that after the extraction with hexane, the insoluble fraction was dried, melted above 270°, and quenched by pouring it into liquid nitrogen. The thus-treated material was finally extracted with boiling toluene.

The ^13^C NMR spectra of the raw material and of the different fractions (Figure S1) show that the fractionation was effective in isolating almost pure polyethylene (Figure S1b, hexane soluble fraction) and syndiotactic polystyrene (Figure S1c, toluene insoluble fraction). However, our attention was devoted to the hexane insoluble-toluene soluble fraction (Figure S1d) whose very simple pattern of signals of the aliphatic region can be safely assigned to the polyethylene sequence at 29.47 ppm and to the syndiotactic polystyrene sequences at 43.85 ppm (T_ββ_, methine carbon) and 40.65 ppm (S_αα_, methylene carbon). The analysis by size exclusion chromatography (SEC) of this polymer fraction revealed a M*_n_* of 56 kDa, while the monomodal shape of the curves and the value of 2.9 for the M*_w_*/M*_n_* ratio supports the idea that we are in presence of a block copolymer (see Figure S5 in the Supplementary Materials).

**Figure 1:**
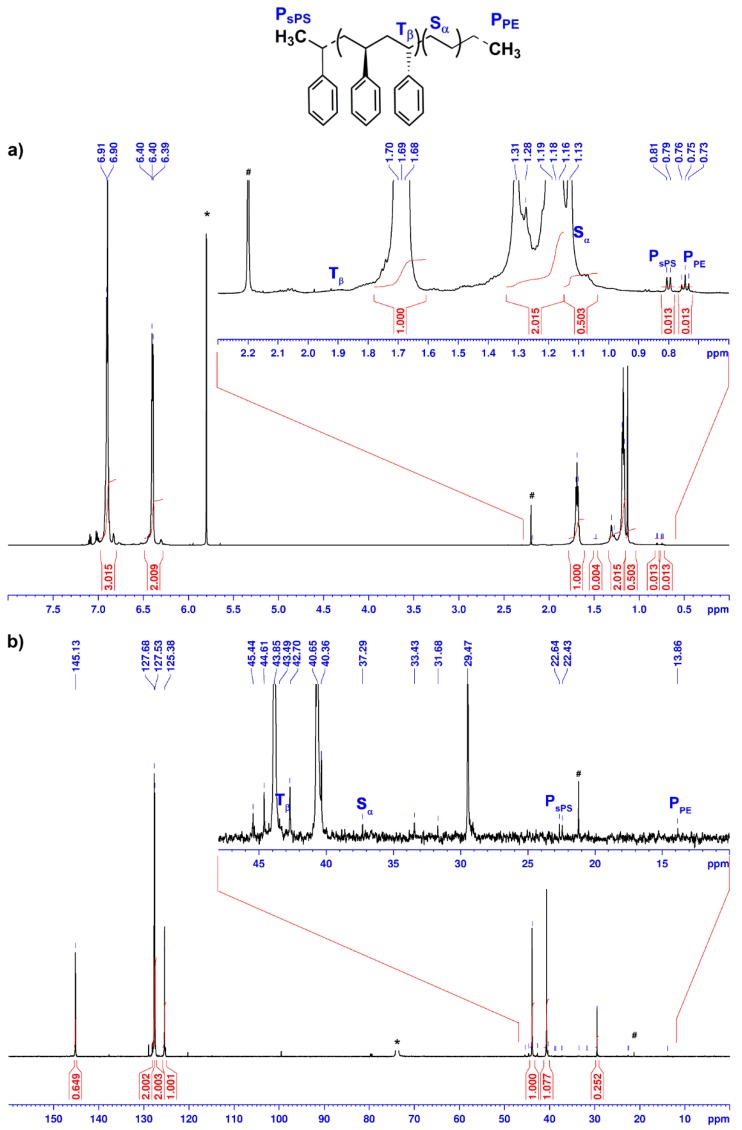
^1^H (**a**) ^13^C spectra (**b**) of sPS-*b*-PE copolymer with magnifications of diagnostic regions for polymer terminals and E-S junction (* TCE-*d*_2_; 70 °C; ^#^ toluene impurity; P, S, and T indicate respectively, primary, secondary, and tertiary alkyl group, while Greek letters indicate the position of the closest tertiary group).

DOSY NMR analysis of the sPS-*b*-PE copolymer has evidenced that the sPS and PE segments have the same diffusion coefficient, providing evidence of the linkage between the homosequences (see Figure S4a). Actually the different dimension of the segments as inferred from the areas of the NMR peaks (S/E molar ratio of 75/25; Figure 1) should imply very different diffusion coefficient if PE and sPS were distinct homopolymers. The molecular weight of 43.8 ± 8.1 kDa, estimated from DOSY NMR diffusion coefficients of sPS-*b*-PE copolymer (9.3 × 10^−12^ ± 2.7 × 10^−13^ m^2^s^−1^) in presence of a PS standard (42.6 kDa; M*_w_*/M*_n_* = 1.04; 9.4 × 10^−12^ ± 2.9 × 10^−13^ m^2^s^−1^), compared well with that determined by SEC (see Figure S4b). 

The hexane insoluble-toluene soluble fraction, ascribed to the sPS-*b*-PE copolymer, was analyzed by ^1^H, ^13^C (Figure 1), two-dimensional ^1^H-^1^H COSY (Figure S2), and ^13^C-^1^H HSQC (Figure S3) NMR spectroscopy, who provided clear evidence for the formation of the diblock architecture. ^13^C NMR analysis of this fraction revealed that the polymer consisted of styrene repeating units with predominant syndio tacticity (diagnostic signal for *rrmrr*, *mrrrr*, *rrrrr,* and *rmrrr* hexades, respectively, at 45.44, 44.61, 43.85, and 42.70 ppm) [32] and linear ethylene repeating units (29.47 ppm), connected by a single (*vide infra*) S-E hetero-sequence (S_α_ at 37.29 ppm; T_β_ at 43.49 ppm; see Figure 1 and Figures S2 and S3) [13,21,23,26,33,34,35]. Resonances centered at 31.68 and 33.43 ppm, associated with regioirregular insertions of S in the PS segment, are also observed to a minor extent [22,36]. The diagnostic signals for isolated styrene units (E-S-E; δ = 44.3, 35.0 and 25.7 ppm) [33,37] or isolated ethylene units (S-E-S; δ = 41.6, 35.5 and 25.1 ppm), as well as those for alternating styrene-ethylene units (44.3, 34.7, 23.4 ppm) [33,36], were not detected. ^1^H NMR chemical shifts of S_α_ and T_β_ signals, for the S-E polymer junction, were identified from ^1^H-^1^H COSY and ^13^C-^1^H HSQC cross-correlations, evidenced, respectively, in Figures S2 and S3, starting from ^13^C NMR signals previously reported [26]. The low molecular weight of the synthesized copolymer also allowed the identification of the NMR signals for the polymer end-groups. The signals at 13.90 and 22.4–22.6 ppm were, respectively, assigned to PE [38] and sPS [22] end groups (P_PE_ and P_sPS_ in Figure 1). ^13^C-^1^H HSQC (Figure S3) and ^1^H-^1^H COSY (Figure S2) cross correlations allowed the identification of the ^1^H NMR signals (Figure 1a) related to the above-described ^13^C NMR signals. It is worth noting that the amounts of polymer terminals and junctions are close to the equimolarity, as can be qualitatively observed from the intensity of the ^13^C NMR signals (Figure 1b), as one could expect for a diblock copolymer consisting of a single polymer junction and two end groups.

It is worth recalling that relevant Ti(IV) reduction took place in these systems with possible oxidation state of +3 in the active sites for syndiotactic styrene polymerization, on the basis of some elegant experimental evidence previously reported [39], As a consequence, the presence of polyethylene (hexane soluble fraction) and syndiotactic polystyrene (toluene insoluble fraction) could be justified by the existence of different oxidation states of Titanium.

### 3.2. Crystalline and Thermal Behavior

**Figure 2:**
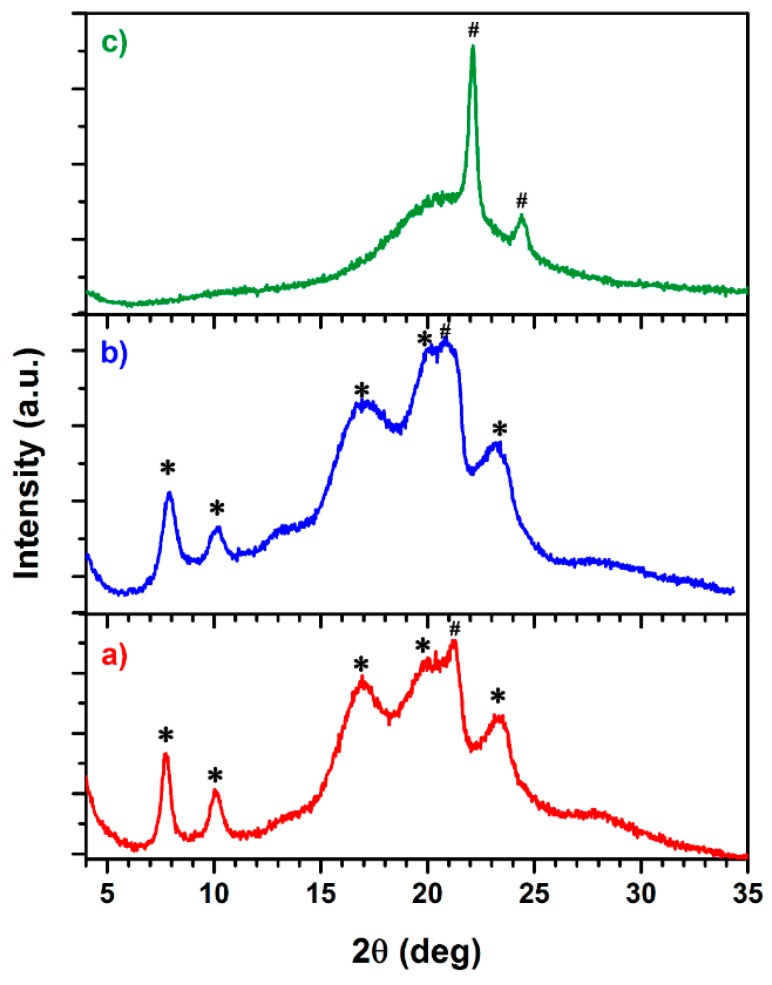
Wide-angle x-ray diffraction (WAXD) patterns for (**a**) crude reaction product, (**b**) of sPS-*b*-PE copolymer, and (**c**) sPS-*b*-PE melted and kept at 270 °C for 20 min then quenched in liquid N_2_. (sPS and PE reflections labelled, respectively, with * and #).

**Figure 3:**
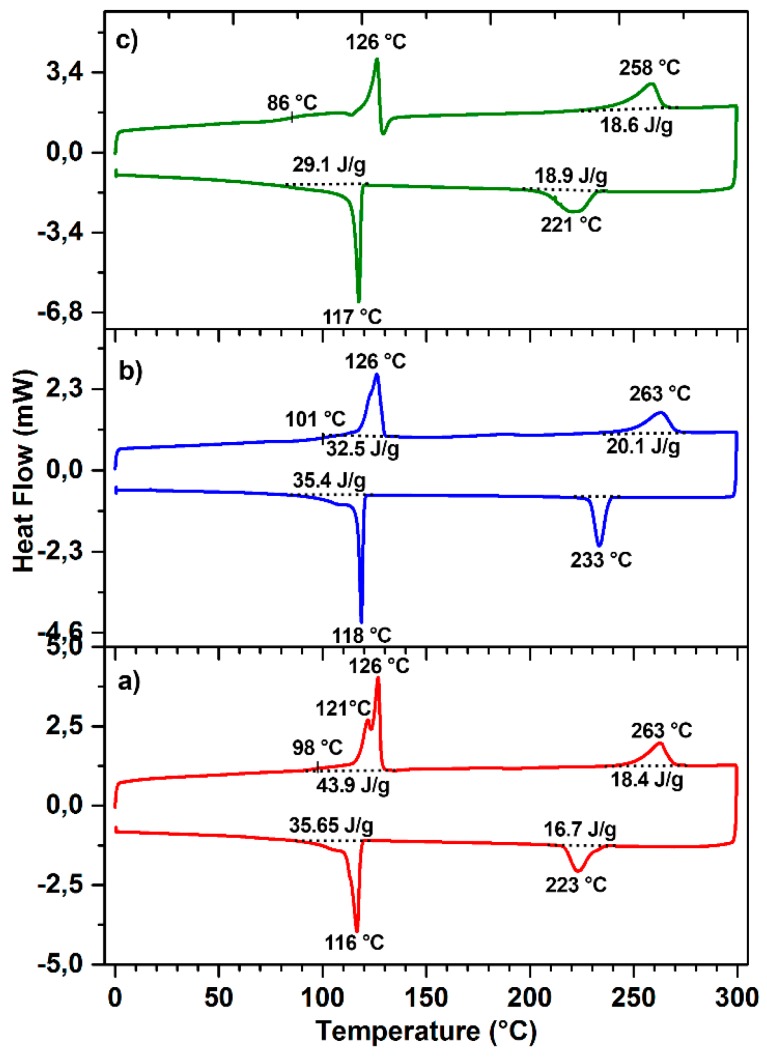
Differential scanning calorimetry (DSC) thermograms for (**a**) raw reaction product, (**b**) sPS-*b*-PE (as recovered after Kumagawa extraction processes), and (**c**) sPS-*b*-PE melted and kept at 270 °C for 20 min then quenched in liquid N_2_.

The copolymer material, as recovered from extraction procedure, shows the x-ray diffraction pattern typical of the δ form of sPS with toluene molecules clathrate in the crystal structure [7,9,40], without relevant signals of the crystallinity related to the polyethylene [41] (Figure 2b). PE crystallinity can be detected in the crude reaction product containing the PE homopolymer (Figure 2a). When the polymer material hexane insoluble/toluene soluble is heated to 270 °C, quickly quenched by pouring it into liquid nitrogen, and kept for some minutes at room temperature, its diffraction spectrum clearly shows the presence of the only crystallinity due to the PE sequence (Figure 2c). The corresponding DSC analysis on such a sample shows an approximate 110 °C overlap of the endothermal peak due to the melting of the polyethylene crystals with the exothermal peak due to the crystallization of the syndiotactic polystyrene in correspondence to its glass transition (Figure 3c). By continuing the heating, one can observe an endothermic phase transition at 255 °C, well below the melting point of the syndiotactic polystyrene, usually reported around 270 °C. Probably, during the crystallization from the solution, the polystyrene can arrange its chains in the usual helices so impeding to the best organization of the polyethylene sequences into the usual lattice of planar *zig-zag* chains. On the other hand, the sample frozen in the amorphous state is able to develop at room temperature the polyethylene crystallinity but is unable to crystallize the polystyrene sequences as long as it is below its glass transition temperature. 

The copolymer preserves the complex polymorphism of the sPS. As an example of this behaviour, the δ-empty form and the β form of sPS can be obtained, respectively, by treatment with supercritical CO_2_ (Figure S6) and by heating the copolymer at 170 °C for 30 min (Figure S7).

### 3.3. Thin Film Morphology

**Figure 4:**
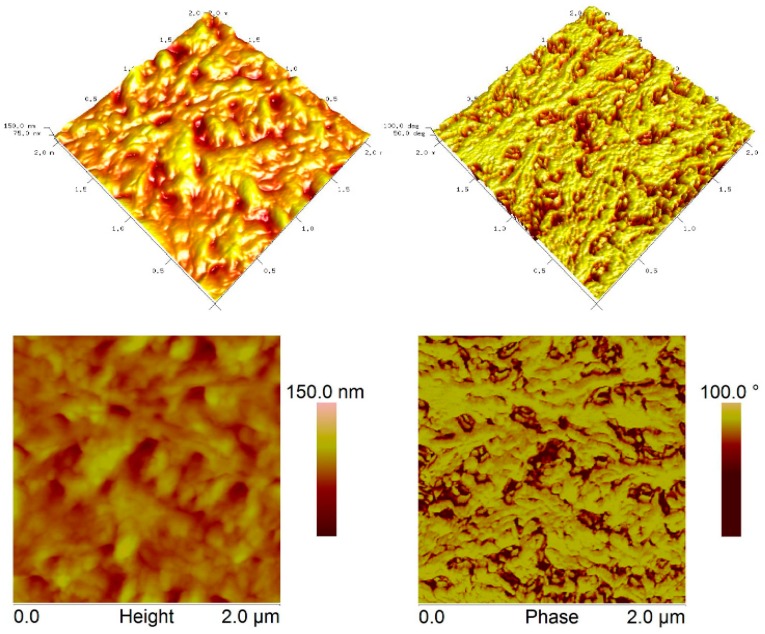
Height (on the left) and phase (on the right) atomic force microscopy operating in tapping mode (TM-AFM) micrographs of thin film of sPS-*b*-PE.

The thin film morphology of the sPS-*b*-PE copolymer has been investigated by TM-AFM. This technique has been widely adopted for the topological characterization of polymer surface as well as for identification of the distribution of the polymer phases on the base of their different mechanical properties [24,42,43,44,45,46,47]. The phase of oscillation of the probe of the AFM nanoscope responds to the stiffness of the surface, allowing the discrimination of chemical phases. Atactic polystyrene (aPS) and PE present quite different stiffness in term of Young’s modulus, respectively, of 2.0 GPa and 0.1 GPa; in fact, they were used as standards for the calibration of AFM nanoscope (operating in HarmoniX or PeakForce mode) for the quantitative determination of mechanical properties of surface [43]. This difference of stiffness is even more pronounced between syndiotactic PS and PE considering the higher modulus of the sPS of 3.0 GPa, w.r.t aPS [43]. The specimens for AFM analyses have been prepared by dissolution of the polymer in TCE (0.2 wt %) at 130 °C and deposition (30 μL) of the polymer solution at 120 °C onto glass slides followed by quenching at room temperature. This procedure allows for the accentuation of the phase separation in these materials, highlighting the eventual presence of homopolymers [43]. The analysis of the thin film prepared with the raw material, prior to the isolation of the sPS-*b*-PE copolymer, presents soft circular regions in micrometre size embedded into a hard matrix, assigned, respectively, to the PE homopolymer and the styrenic counterparts (see Figure S8). The thin films of the polymer material coming from the fractionation procedure (S/E weight ratio = 75:25) present a smooth surface, from the phase contrast analysis found to consist of a hard matrix with incorporated a soft jagged phase with nanometre scale. In this case, the PE segregates to form small nanometric domains, because it is bounded to the sPS segments (Figure 4). 

## 4. Conclusions

An ethylene-styrene block copolymer with a syndiotactic styrene sequence joined to the polyethylene chain can be obtained, together with sPS and PE homopolymers, by using the classic catalytic system CpTiCl_3_/MMAO through sequential polymerization of the monomer at a low temperature. The mixture can be resolved for isolating the sPS-*b*-PE copolymer via consecutive solvent extraction of the raw polymer material through the Kumagawa apparatus. Our success in obtaining such a copolymer with a long ethylene sequence can be ascribed to the low reaction temperature that protracts the lifetime of the propagating chain by lowering the transfer and termination rate. 

The in-depth characterization of the new material through size exclusion chromatography, differential scanning calorimetry, and various NMR techniques supports this block architecture. In addition, it has been observed that the sPS-*b*-PE copolymer substantially preserves the crystalline and thermal behaviour of the two polymer segments, in particular, the well-known nano-porosity of the syndiotactic segment. The analysis of thin film of the sPS-*b*-PE copolymer by TM-AFM evidenced a phase-separated morphology with hard polymer matrix of sPS and embedded jagged nanometric soft domains of PE. Finally, this material possibly can be seen as a new member of the short list of the nanometric polymeric sieves, and this perspective will be explored in future research activity.

## Supplementary Materials

The following are available online at https://www.mdpi.com/2073-4360/11/4/698/s1, Further details on NMR, SEC, WAXD, and TM-AFM analyses.
