# Supplementary material for: Synthesis and Characterization of Syndiotactic Polystyrene-Polyethylene Block Copolymer
Source: Polymers (Basel). 2019 Apr 16;11(4):698. doi: 10.3390/polym11040698 (PMC6523272; doi:10.3390/polym11040698)
Supplement: Supplementary file 1 [file polymers-11-00698-s001.pdf]

Synthesis and Characterization of a Block Copolymer Syndiotactic Polystyrene-*b*-Polyethylene

David Hermann Lamparelli [1], Vito Speranza [2], Isabella Camurati [3], Antonio Buonerba [1,*] and Leone Oliva [1,*]

[a] Dipartimento di Chimica e Biologia "*Adolfo Zambelli*", Università degli Studi di Salerno, via Giovanni Paolo II, 84084 Fisciano (SA), Italy.

[b] Dipartimento di Ingegneria Industriale, Universit àdegli Studi di Salerno, Via Giovanni Paolo II, 84084 Fisciano (SA), Italy.

* Correspondence: A.B.: abuonerba@unisa.it and L.O. loliva@unisa.it

Table of Contents

1. NMR Analysis......S3

Figure S1. ^{13}C NMR spectra (*TCE-d_2; 70 ℃) of polymer fractions deriving from the synthetic procedure adopted for the isolation of sPS-*b*-PE copolymer: a) crude reaction product; b) hexane soluble fraction; c) toluene insoluble fraction; d) toluene soluble fraction. S3

Figure S2. ^{1}H-^{1}H COSY NMR spectrum of sPS-*b*-PE (a) with magnification (b) of the diagnostic region for polymer terminals and E-S junction (* TCE-d_2; 70 ℃; # toluene trace impurity)......S4

Figure S3 ^{1}H-^{13}C HSQC NMR spectrum of sPS-*b*-PE (a) with magnification (b) of the diagnostic region for polymer terminals and E-S junction (* TCE-d_2; 70 ℃; # toluene trace impurity)......S5

Figure S4. DOSY NMR spectra of: a) sPS-*b*-PE; b) sPS-*b*-PE (diffusion coefficient = $9.3 \cdot 10^{-12} \pm 2.7 \cdot 10^{-13}$ m^2s^{-1}) in presence of a PS standard (42.6 kDa; M_w/M_n =1.04; diffusion coefficient = $9.4 \cdot 10^{-12} \pm 2.9 \cdot 10^{-13}$ m^2s^{-1}). S6

2. SEC Analysis S7

Figure S5. SEC curves the polymer fraction: a) soluble in hexane; b) insoluble in toluene; c) soluble in toluene (* front of solvent)......S7

3. WAXD Analysis......S8

Figure S6. WAXD diffractogram of the sPS-*b*-PE copolymer after extraction with supercritical carbon dioxide......S8

Figure S7. WAXD diffractogram of the sPS-*b*-PE copolymer after treatment at 170 ℃ for 30 min. S8

4. TM-AFM Analysis S9

Figure S8. Height (on the left) and phase (on the right) TM-AFM micrographs of thin film crude reaction product coming from the synthetic procedure of sPS-*b*-PE copolymer......S9

1. NMR Analysis

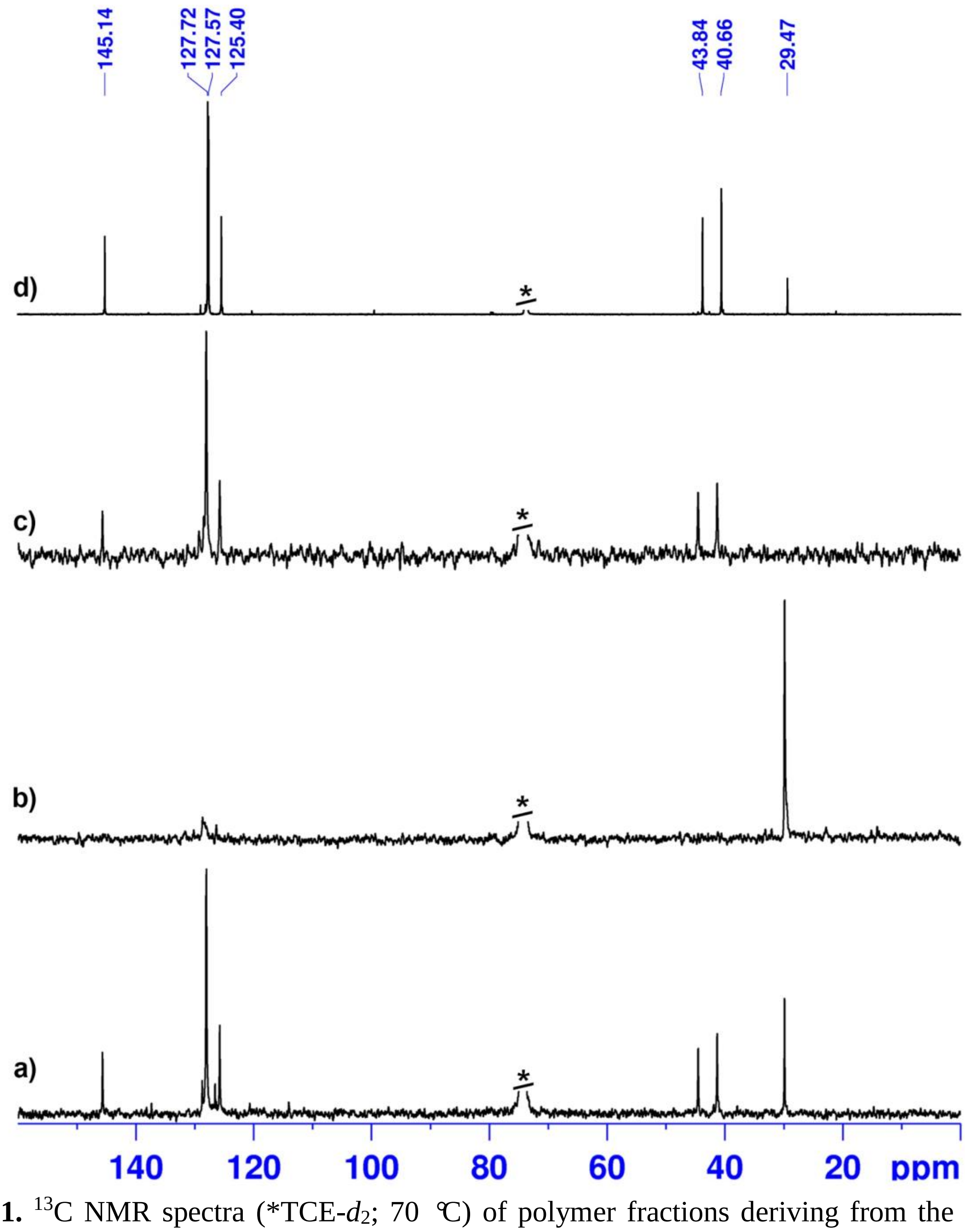

Figure S1. ^{13}C NMR spectra (*TCE-d_2; 70 °C) of polymer fractions deriving from the synthetic procedure adopted for the isolation of sPS-*b*-PE copolymer: a) crude reaction product; b) hexane soluble fraction; c) toluene insoluble fraction; d) toluene soluble fraction.

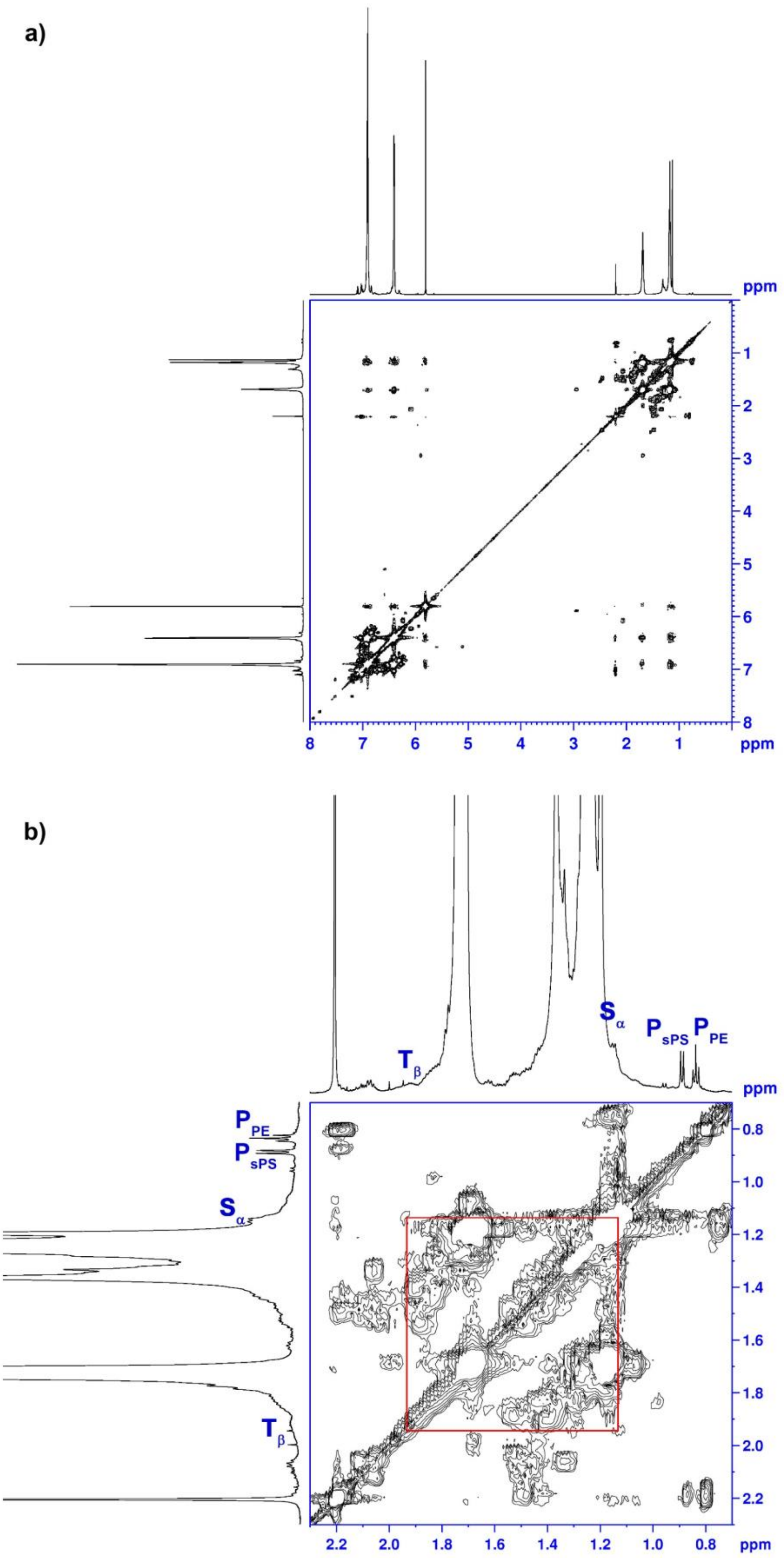

Figure S2. ^{1}H-^{1}H COSY NMR spectrum of sPS-*b*-PE (a) with magnification (b) of the diagnostic region for polymer terminals and E-S junction (* TCE-d_2; 70 ℃; # toluene trace impurity).

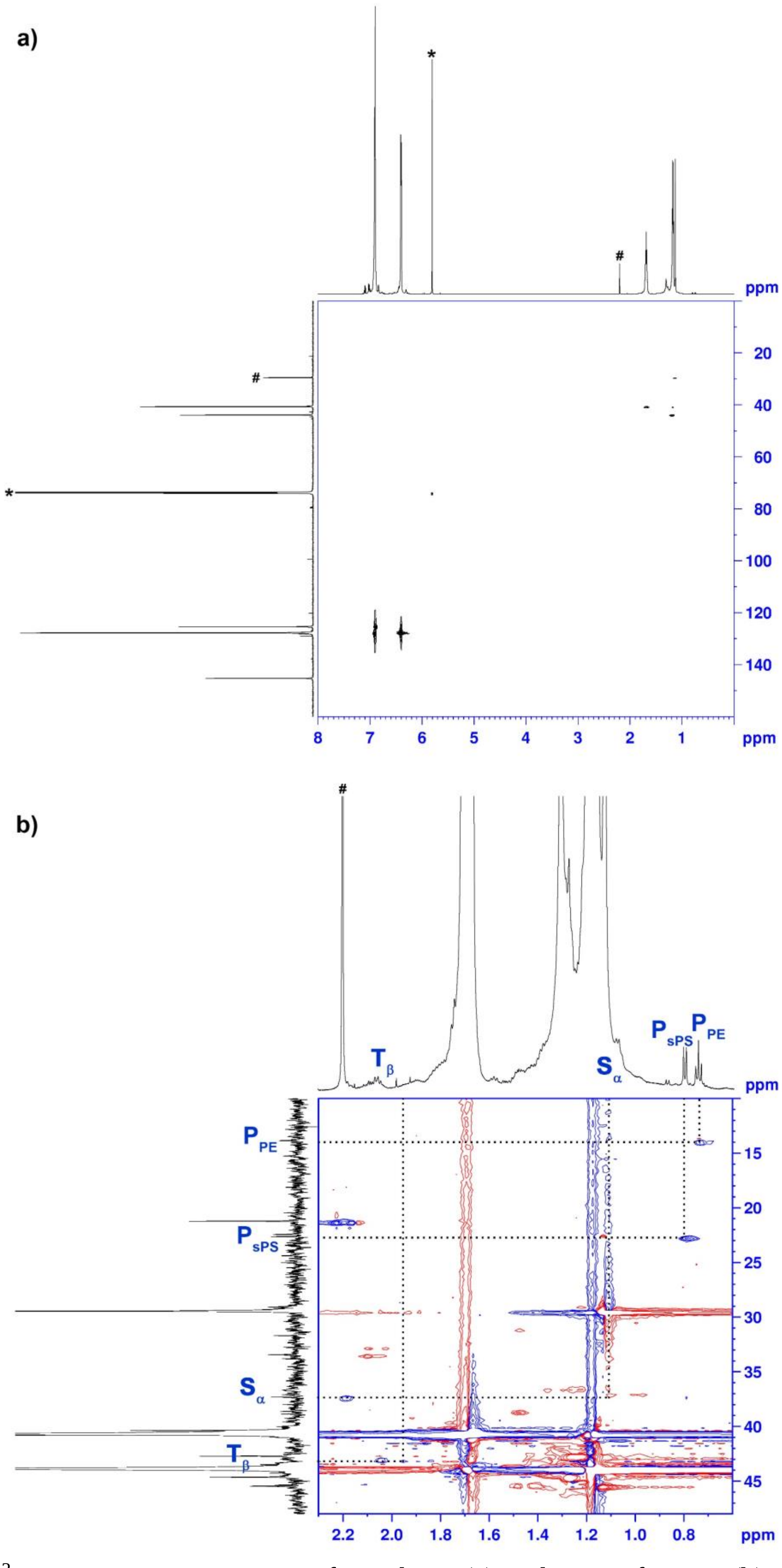

Figure S3 ^{1}H-^{13}C HSQC NMR spectrum of sPS-*b*-PE (a) with magnification (b) of the diagnostic region for polymer terminals and E-S junction (* TCE-d_2; 70 ℃; # toluene trace impurity).

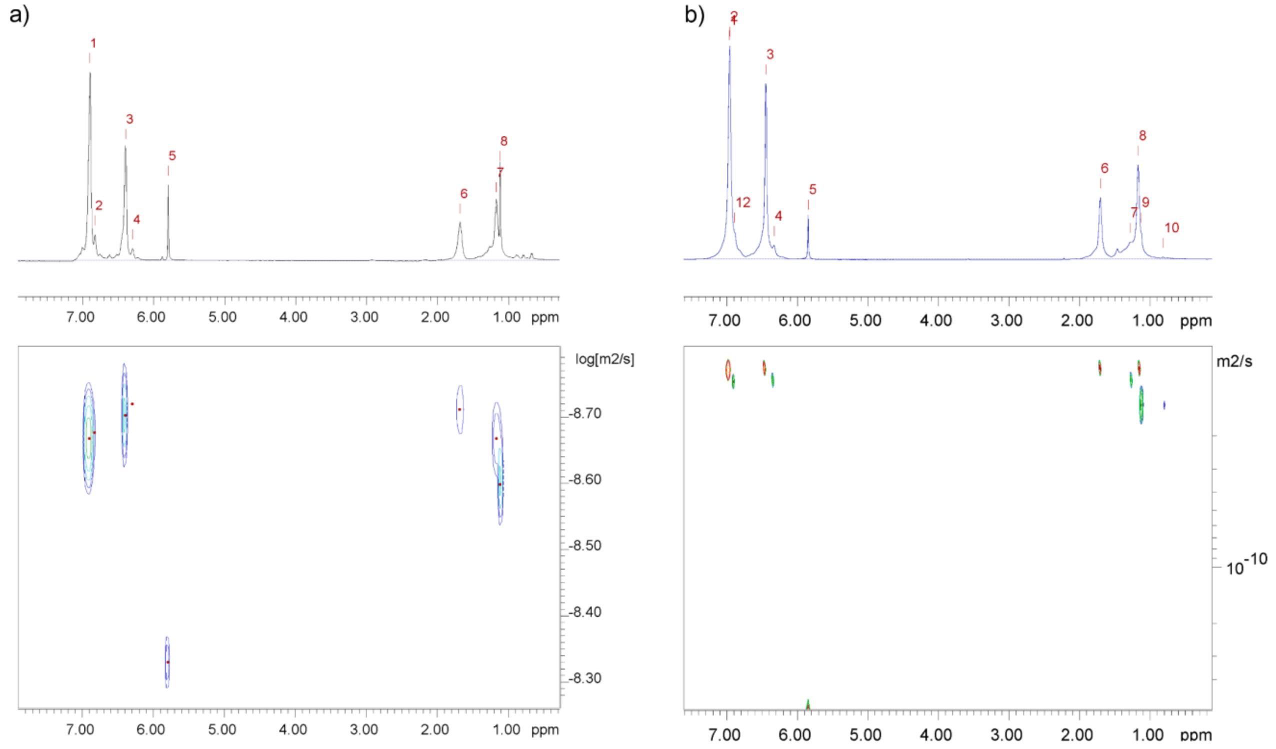

Figure S4. DOSY NMR spectra of: a) sPS-*b*-PE; b) sPS-*b*-PE (diffusion coefficient = $9.3 \cdot 10^{-12} \pm 2.7 \cdot 10^{-13}$ m^2s^{-1}) in presence of a PS standard (42.6 kDa; M_w/M_n =1.04; diffusion coefficient = $9.4 \cdot 10^{-12} \pm 2.9 \cdot 10^{-13}$ m^2s^{-1}).

2. SEC Analysis

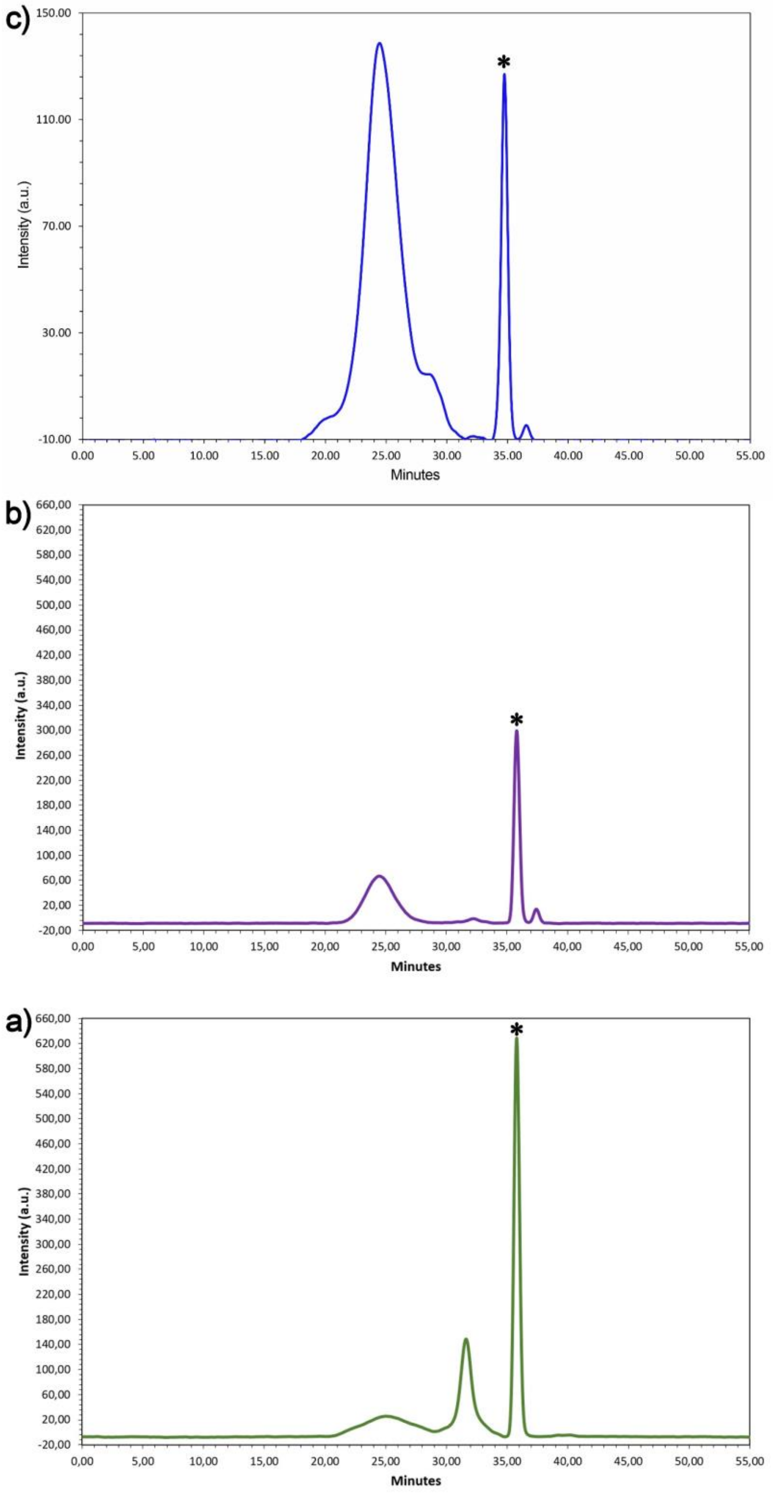

Figure S5. SEC curves the polymer fraction: a) soluble in hexane; b) insoluble in toluene; c) soluble in toluene (* front of solvent).

3. WAXD Analysis

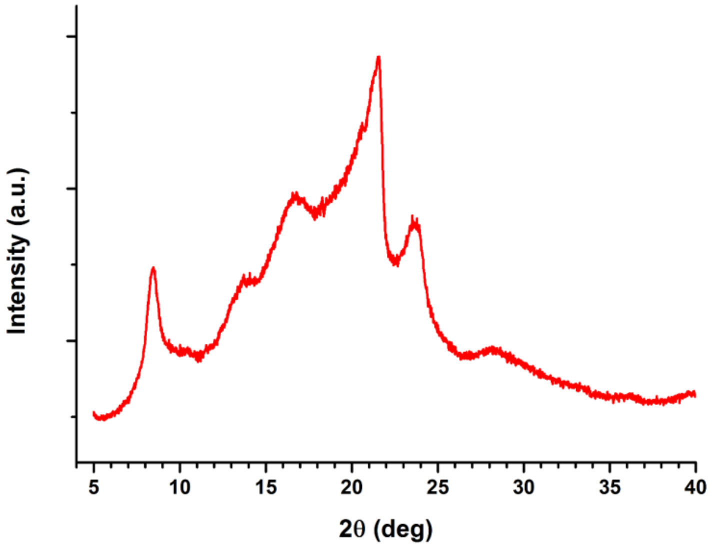

Figure S6. WAXD diffractogram of the sPS-*b*-PE copolymer after extraction with supercritical carbon dioxide.

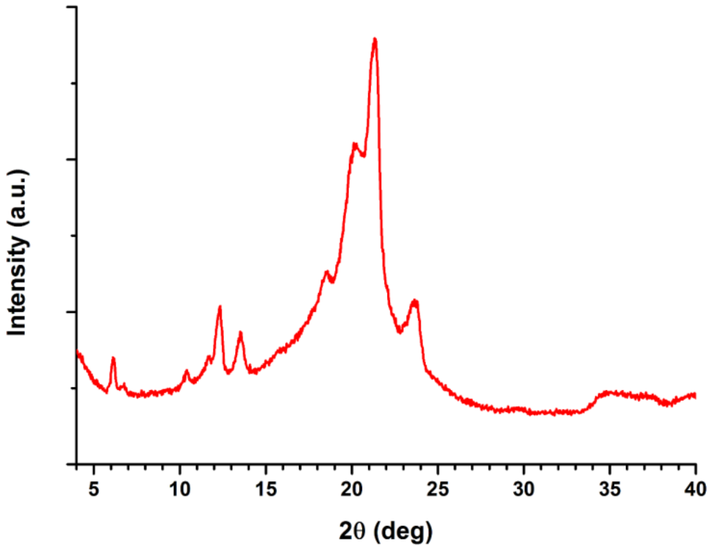

Figure S7. WAXD diffractogram of the sPS-*b*-PE copolymer after treatment at 170 ℃ for 30 min.

4. TM-AFM Analysis

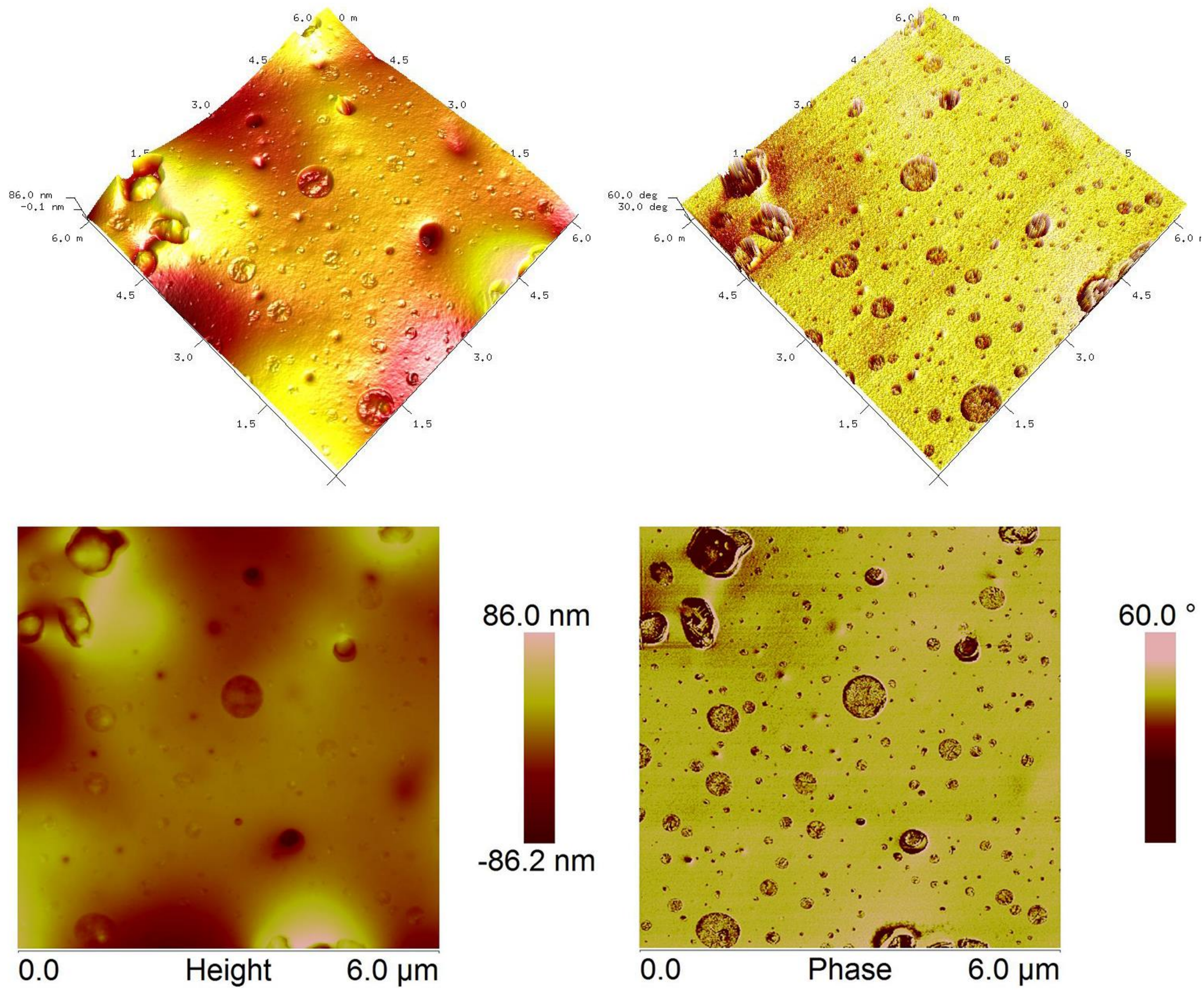

Figure S8. Height (on the left) and phase (on the right) TM-AFM micrographs of thin film crude reaction product coming from the synthetic procedure of sPS-*b*-PE copolymer.
